# A Talk about Voluntary Hospitals
*At St. James's Palace to the annual meeting of the League of Mercy. See p. 286


**Published:** 1915-12-25

**Authors:** 

**Affiliations:** Lord Chamberlain, G.C.I.E., G.C.S.I.


### A TALK ABOUT VOLUNTARY HOSPITALS.*

BY THE RT. HON. LORD SANDHURST, G.C.I.E., G.C.S.I. (LORD CHAMBERLAIN).

May I, your Royal Highness, with great respect, congratulate you not only upon this year's work, but on the aggregate work of the League of Mercy? I am a collector myself, and have been for many years, and the worse the time seems to be, the more deeply friends seem willing and anxious to put their hands into their pockets to help us. In the various institutions that I have to do with, of course, we feel the strain of the increased price of commodities, but our subscribers have in no way fallen off, and if there is one way in which the people can help the war more than another, those who cannot actively assist in it, it is by taking care of those who have been doing so much for the country without a murmur, and with enthusiasm in its hour of need.

### The House of Lords Committee.

Thirty years ago I presided over a Committee which had for its object the inquiry into the whole of the Metropolitan Medical Relief. We had before us, as a rule, the officials, and certainly all their reports and accounts, of all the hospitals, I should say, of London. And what impressed us all so very much was that the management of these institutions might be said, without fear of contradiction, to be universally good. And not only that, but the work of the managers themselves was unremitting and disinterested to the last degree, without any suspicion on their part of any axe to grind. Having had a great deal to do for thirty years with the voluntary hospitals, I have never been one of those who were rather afraid that the State might step in and take our property from us and take over our patients. There were two reasons for that mainly; one was that the State has plenty to do to look after itself. The other was that beyond a few critics, who no doubt stimulate us and do us good, the hospitals themselves are well managed; all those who seek the hospitality of their walls are looked after by the doctors, surgical and medical, and by the nurses with a tenderness and care which it is impossible to surpass. They are also the great halls for teaching the rising generation of medical men; they are the real and genuine civilising influences which are inevitable for all of those who come within their walls, and there is in them the very best and highest knowledge, combined with all the skill that the advances of science can give. All that is at the disposal of every patient, whatever his degree or her degree may be. And I have every confidence that it will go on, and I believe the position of the voluntary hospitals is unassailable.

### The National Insurance Act.

I remember very well the Insurance Act. I remember that a great many pessimists, or as I prefer to call them—over-anxious people—suggested that the knell of the voluntary system had sounded. But it was not what in reality happened. Of course, from time to time new ideas, even within the walls of hospitals, supervene; the populations increase, progressive circumstances develop, and legislation of various descriptions is passed; but the first thing we did at the passing of the Insurance Bill was to take counsel with the medical men on our staff; that is to say, the administrative and expert side, to see how we could best turn the Insurance Bill to account. The result was that with a very little patience and painstaking, we got hold of methods which resulted in at any rate one department of hospitals benefiting considerably by the passing of that Insurance Act.

### Are Hospitals Abused?

I have been told quite frequently that owing to the voluntary system being in some directions rather lax—I would prefer to put it as possibly over sympathetic—hospitals are liable to abuse—that is to say, that they are used both in the out-patients' and the in-patients' departments by those who can afford to go their own doctors. There, again, an experience of over thirty years or more has not allowed me to subscribe to that doctrine. And another thing is that it is extremely difficult, unless you are really an expert, which I do not claim to be for a moment, to know who is a fit person for charity, or be able to condemn one who looks as if he were an unfit person for charity. It is acknowledged by those who know anything of the subject that a comparatively well-made coat or skirt is no guide whatever to the circumstances of the wearer. We know that in certain alleys and small narrow streets there are coats and skirts which go the round for special occasions, from person to person, and we must remember that poor people have their pride. A poor person wants to go to the hospital for his occasional visit, and this coat is donned for the occasion. In that way the resident from that humble locality seeks to present as respectable an appearance as possible when entering the waiting-rooms. That will show how difficult it is to know who are fit for relief.

### A Useful Guide.

But there is one way in which I have been able to form an opinion as to whether hospitals are abused or not. We all know there are visiting days at these big hospitals, and if you go through the wards on those occasions and carefully observe the many friends of the patients, I think your observation will lead you to the conclusion that those friends who are visiting are not in a position to help themselves. Of course, another safeguard is, as people who know hospitals are aware, that the hospital is per-

* At St. James's Palace to the annual meeting of the League of Mercy. See p. 286.

forming a work of charity and that it is not a rate-aided institution to which, therefore, any member of the public who pays rates has the right of entrance. There is the further safeguard that by tactful inquiry of suspicious-looking people they may be found to be imposing. Therefore, I think it is safe to say that the hospitals are not abused.

### Nurse Cavell and British Nurses.

I mentioned just now about the training of the younger generation. But what about the nurses of whom thousands are now in France and at the various theatres of war by land and sea? They all got their training in these voluntary hospitals, or at any rate the vast majority of them did, and I would call to mind a name which has immortalised its possessor, Nurse Cavell, not more immortalised by the murderous brutality of her end than by her heroic and gentle disposition in all the work she had to do, the product of a voluntary hospital training, of her work in that admirable institution which we all know so well, the London Hospital, which is presided over by our dear old friend whom we used to know as The Honourable Sydney Holland, and who is now Lord Knutsford.

### The League of Mercy and its Work.

The League of Mercy, and any other collecting institutions, are interdependable one on the other. The voluntary system as a whole depends on the League of Mercy and other collecting bodies for the funds with which to carry on its work. You on your side have a right to see that the money is properly expended, that there is no waste, and that efficiency is properly maintained. We all know the shifts we are put to to collect money; at any rate I do. We know the terrible and ingenious methods by which we try to get money from our friends, and, I must say, with considerable success. Think of the bazaars, the sales, the various fêtes with side shows to entrap the unwary by another half-crown, the cups of tea at 2s. 6d. and 10s. each because they have been tasted by some beauty. But where your institution has beaten us all in its working is that while we have had our dinners and squandered thousands of pounds in this and that direction—and nobody knows this better than the Rt. Hon. the Lord Mayor—the League of Mercy has, with its various ramifications, its organisation, and so on, touched what I venture to call the shilling and half-crown side of charity, and on the principle of the snowball those little modest sums which are marked on these cards, the pence, the three-penny pieces, the sixpences and shillings have grown up into this aggregate, to a quarter-of-a-million pounds in these years. But I am extremely obliged to my old friend of so many years' standing for another hint which he has given me to-day and which I am bound to say never occurred to me, and that is the film. It seems to me there is a great deal in it, and I shall go to my directors and advisers at St. Bartholomew's Hospital and ask them what they think about it.

### The Royal Family of England.

My heart is full of this voluntary system. But there is no one family among all its members who has done more for the voluntary system than has the Royal Family of England. By the gracious interest, by the visits which are so much welcomed, there is hardly an institution which has not been visited by them. His Serene Highness, your Grand President, until he went to the Front, was Chairman of the Middlesex Hospital Board. His brother before him was an enthusiastic worker and Chairman of the same hospital; and they show us the fine example of persuasion and tact which is the basis of the sympathy which pervades everything which has to do with a general hospital.

### A Story about Sympathy.

I would like to tell you a story about sympathy, first because it is true, and secondly, because it is a remarkable instance of what a young sympathetic person can do. When I was in India twenty years ago plague broke out, and the Secretary of State sent me out fifty to sixty young medical men who had lately qualified. I was sent for to a distant part of the Presidency, which I had the honour to preside over, because there were some difficulties with a portion of the Indian population. In fact, what we could not get them to do was to move, practically, from one side of the road to the other. I went down to this place and I was met by the chief officer of the district. He said, "We are very glad to see you, sir, but we are sorry to have brought you here because the difficulty is at an end." I said, "I am very glad to hear that; it makes the visit all the nicer. But how was it accomplished and who accomplished it?" The answer was, "The young man you sent from Bombay." I asked, "Where is he?" He was brought, and I complimented him and said, "Now, Mr. So-and-so, you have done extraordinarily well, how did you do it?" The young man came from Scotland and he was a man of few words. He said, "I don't know." I said to him, "You talk Hindustani?" His answer was "No." "What do you talk?" "I talk English." "But you got them all out?" "Yes." Now that young man, who could not talk a word of the language and who was as unintelligible to the natives as the natives were to him, somehow or another by that electric current which we call sympathy from man to man managed without the least difficulty to get those people out of their infected areas into the other area, and the work was done. I think he came from University College Hospital. That is an instance of the teaching and bringing up in one of the hospitals, combined with the sympathetic nature of that work.

Now, your Royal Highness, I have once more in conclusion to congratulate you on the admirable work which you have done as the lady Grand President. May I also, with great respect, express the hope that these labours which have been carried on with so much energy and success will, as I feel confident they will, be equally energetically pushed, and to even greater successes, in the future?

